# The Impact of Subjective Poverty on the Mental Health of the Elderly in China: The Mediating Role of Social Capital

**DOI:** 10.3390/ijerph20176672

**Published:** 2023-08-29

**Authors:** Yanghan Li, Tianyi Chen, Quan Li, Linxiu Jiang

**Affiliations:** 1 School of Political Science and Public Administration, Wuhan University, Wuhan 430072, China; 2015lyh@whu.edu.cn (Y.L.); linxiu@whu.edu.cn (L.J.); 2 Institute of Quality Development Strategy, Wuhan University, Wuhan 430072, China; tianyichen@whu.edu.cn

**Keywords:** subjective poverty, mental health, social capital, the elderly, CLHLS

## Abstract

Reducing subjective poverty among the elderly is an important aspect of poverty governance and is a necessary part of implementing the healthy aging strategy in China. In both China and the majority of low- and middle-income countries, systematic research on the relationship between subjective poverty and the mental health of the elderly needs to be expanded. In this study, we aimed to examine how social capital, including bonding and bridging social capital, mediate the relationship between subjective poverty and mental health among the elderly in China. Relying on the 2018 Chinese Longitudinal Healthy Longevity Survey (CLHLS) data, we used ordered probit (oprobit) regression, propensity score matching (PSM), and instrumental variable (IV) regression to estimate the effects of subjective poverty on mental health. The results indicate that subjective poverty has a significant negative impact on the mental health of the elderly in China. More importantly, social capital, including bonding and bridging social capital, partially mediates the relationship between subjective poverty and mental health. We believe that, in the process of implementing the government’s healthy aging strategy in China, society and government should recognize the importance and value of subjective poverty governance for the elderly. In particular, the construction of a social relationship network that centers on bonding and bridging social capital could be instrumental in dealing with subjective poverty among the elderly and safeguarding their mental health and wellbeing.

## 1. Introduction

Mental health is not only a perplexing public health challenge, but also a complex social governance issue. According to the *World Mental Health Report: Transforming Mental Health For All* released by the World Health Organization (WHO) in June 2022, one billion people and 15% of the adult workforce suffered from mental health disorders in 2019 [1]. Mental health is an important human right that is related to individuals’ dignity and wellbeing. It is generally believed that individuals are more likely to experience mental illness when they are in an unfavorable environment [2]. As one of the most vulnerable and disadvantaged groups, the elderly are more likely to be subject to the risk of mental health. Factors such as a decline in physiological function, various acute and chronic stressors in the environment, and economic and social vulnerabilities could have a negative impact on the daily lives of the elderly, resulting in increased psychological stress and a decline in mental wellbeing [3,4]. Many studies have shown that poverty is one of the main factors leading to mental health disorders in the elderly [5,6,7].

China is a country with a large number of elderly people and has transitioned into a moderately aging society. The data released by the Chinese government statistical department show that the number of people aged 65 and over has reached 209.8 million, accounting for 14.86% of the total population by the end of 2022 [8]. According to the *White Paper on the Mental Health of the Elderly in China*, 95% of this population suffer from some degree of a mental health disorder [9]. It took China decades to wind down its anti-poverty campaign. By the end of 2020, absolute poverty, which is measured in terms of basic living needs, was finally eliminated. Nevertheless, due to factors such as personal ability, social division of labor, and resource endowment, there still exist huge economic and social inequalities in China [10], which inevitably lead to perceptions or emotions of unmet needs, relative deprivation, and a low life-satisfaction level among the elderly [11], resulting in subjective poverty. Existing studies have shown that subjective poverty often acts a more significant role in physical and mental health and personal wellbeing than objective poverty [12,13].

In low- and middle-income countries, studies on the poverty and wellbeing of the elderly have often focused more on the effects of objective poverty on individuals’ health. Little is known about the relationship between subjective poverty and the mental health of the elderly. In particular, the underlying mechanism between subjective poverty and mental health has not been closely examined in the literature. In this study, we explore the effect of subjective poverty on the mental health of the elderly, as well as the underlying mechanism of social capital (including bonding and bridging social capital) in the relationship between subjective poverty and mental health. The results of this study can help to improve anti-poverty policies, enhance awareness of the importance of the social relationship network among the elderly, and ensure their mental health and wellbeing in the new era.

## 2. Literature Review

### 2.1. Subjective Poverty and Mental Health

In recent years, the impact of subjective poverty on mental health has gradually attracted the attention of researchers. Subjective poverty involves the subjective assessment and judgment made by individuals that their needs cannot be met. This is the result of the subjective mapping of individual or family economic and social conditions [14,15] and is also closely related to the reference group [14,16,17]. Pearlin’s stress process model can be used to explain the effect of the stress caused by subjective poverty on mental health [18]. As a source of stress, subjective poverty causes individuals to gradually lose control of their lives, generating feelings of frustration, self-blame, and loss of self-efficacy, which eventually leads to mental health issues [19]. As pointed by the social comparison theory, people often assess themselves by comparing themselves to others and upward comparison usually leads to perceived deprivation, while downward comparison leads to higher self-esteem [10].

Studies have found that subjective poverty is marked by psychological stress [20,21], depression, and loneliness [22], thereby reducing individuals’ happiness, quality of life, and self-rated health [23]. Some studies have stated that the stress and feelings of social deprivation caused by subjective poverty can hinder individuals’ social participation and integration, making it difficult for them to solicit adequate resources from the surrounding environment, which will reduce the individuals’ future expectations and life satisfaction [24,25]. A study on Hong Kong residents confirmed a significant correlation between subjective poverty and mental health. The researchers found that, compared with reducing objective poverty, reducing subjective poverty may be more helpful in improving residents’ mental health [26]. The latest research has also confirmed the significant impact of subjective poverty on mental health; namely, subjective poverty is a powerful predictor of mental health and quality of life [24]. However, to our knowledge, most of the studies that examine the relationship between subjective poverty and mental health have focused on adolescents and middle-aged people and have barely focused on the elderly in low- and middle-income countries.

### 2.2. Subjective Poverty, Social Capital, and Mental Health

Social capital is an ambiguous concept that is difficult to define, and there is diversity in the definition, dimensions, and measurement in the literature [27]. It is usually regarded as a social network with relatively stable, institutionalized, and sustainable characteristics [28]. In mental health research, while many studies have proposed that social capital is of vital significance [29,30,31], there is generally little empirical evidence that sheds light on the relationship between social capital and mental health [32]. Some scholars have noted that there is uncertainty in social network support for mental health, and different social capital practices affect mental health in different ways [21]. For example, in Nyqvist et al.’s view, social capital is described as an umbrella concept that encompasses all aspects, types, and levels of social resources, with various meanings, and its relationship with mental health is hard to predict [33]. Ehsan and Silva concluded that the role of social capital and its relationship with mental health differ in various contexts [34]. Generally speaking, the effect of social capital on mental health rests with its definition, dimensions, and measurement [35], as well as the specific context [36].

As for the impact of social capital on the mental health of the elderly, Haseda et al. argued that social interaction and engagement are key aspects of the social capital of the elderly, and that social support, trust, and the sense of belonging obtained in this process are crucial to mental health and wellbeing [37]. Some studies have divided social capital into cognitive social capital and structural social capital and found that cognitive social capital can reduce depressive symptoms in the elderly [38,39], but the impact of structural social capital on the mental health of the elderly is controversial due to regional and cultural differences [36]. Some studies have divided social capital into multiple dimensions, such as rules, trust, and community partnerships, and have confirmed that the impact of different dimensions of social capital on the mental health of the elderly depends on ethnicity [40]. Nevertheless, when it comes to the elderly in low- and middle-income countries, few scholars have examined the impact of social capital on their mental health by differentiating between bonding and bridging social capital [41,42].

As previous studies have demonstrated, social capital functions as a linking mechanism between poverty and mental health [26,43,44]. When individuals are in stressful situations generated by poverty, they might seek support from family and society [45], which includes emotional support, integration, participation, and opportunities [46]. Social capital, as an embedded resource that can be obtained from social networks [27], can provide the help, support, resources, and services needed by the elderly [47], relieving negative sentiment, for instance, depression, loneliness, and anxiety [48], thereby improving their physical and mental health. A recent study found that the psychological stress and negative emotions generated by subjective poverty can be buffered by various resources provided by social capital [4]. Nevertheless, little research has been done to explore the mediating role of social capital in the relationship between the subjective poverty and mental health of the elderly.

### 2.3. Current Study

**Figure 1:**
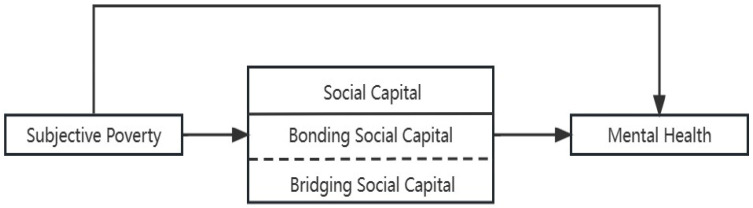
Conceptual framework.

To reiterate, while the existing research has already explored the relationship between subjective poverty and mental health, few scholars have examined the elderly in low- and middle-income countries. More importantly, studies that consider the mediating effect of bonding and bridging social capital on subjective poverty and mental health are rare. Unlike that of high-income countries, the economic and social development of low-and middle-income countries is generally unbalanced; the development of organization-based social capital is not sufficient, and their social capital is more reflected in informal interpersonal networks. As a developing country, China shares many traits with other low- and middle-income countries. There is a large gap in the levels of economic and social development between urban and rural areas in China. In addition, organization-based social capital is less prevalent in China than in Western developed countries. China’s culture typically attaches great importance to the relationships among family members, and society emphasizes personal networks that are based on particularistic relationships and interpersonal interactions [42]. Therefore, we sought in this paper to examine the relationship between subjective poverty and mental health among the elderly in China. Particularly, we consider the mediating effect of individual-level social capital, i.e., bonding and bridging social capital. Figure 1 shows the conceptual framework, and the associated hypotheses are presented below:

**Hypothesis 1 (H1).**  *Subjective poverty has a significant negative impact on mental health among the elderly in China.*

**Hypothesis 2 (H2).**  *Social capital partially mediates the relationship between subjective poverty and mental health among the elderly in China.*

**Hypothesis 2a (H2a).**  *Bonding social capital partially mediates the relationship between subjective poverty and mental health among the elderly in China.*

**Hypothesis 2b (H2b).**  *Bridging social capital partially mediates the relationship between subjective poverty and mental health among the elderly in China.*

## 3. Materials and Methods

### 3.1. Data

We used data from the Chinese Longitudinal Healthy Longevity Survey (CLHLS) 2018. The CLHLS is a scientific research project jointly conducted by Peking University and the Chinese Center for Disease Control and Prevention (CDC). It is the earliest and longest-lasting special sampling survey in the field of aging research in China. The survey aims to obtain health information among the elderly, aged 65 years and over, so that academics and policy makers can better understand the influencing factors of health in the elderly population. In our study, we selected the 2018 data of the survey for analysis. The 2018 survey covered more than 500 sample points in 22 provincial administrative regions, with a total of 15,874 respondents; these data can generally reflect the basic situation of China’s elderly population. 8811 valid samples were retained after screening and excluding invalid observations.

### 3.2. Variable Selection

#### 3.2.1. Outcome Variable

The outcome variable of the study was mental health. Depression is one of the key indicators of mental health and is often used to measure an individual’s mental health status. Following common practice in the literature, we used depression indicators to measure the mental health of the elderly [49,50,51]. The CLHLS 2018 questionnaire used the Center for Epidemiological Studies Depression Scale (CES-D) to measure depression, and it contained eight negative items and two positive items. We used a reverse-scoring operation on the two positive items worded as, “I am hopeful for the future” and “I am happy”. Each item in the CES-D contains five options—“always”, “often”, “sometimes”, “rarely”, and “never”—and we combined the options “sometimes” and “rarely” into the potion “sometimes or rarely”. In terms of the variable assignment, “never”, “sometimes or rarely”, “often”, and “always” were recorded as 0 to 3, respectively. The final scores ranged from 0 to 30, with a higher score indicating a higher degree of depression and a lower level of mental health. According to Wu et al. [52], we took scores at 10 and 14 as the critical values for dividing mental health status, and then we recorded the scores of the respondents. Scores of 15–30, 10–14, and 0–10 were recorded as 1 to 3, respectively, corresponding to “unhealthy”, “not very healthy”, and “healthy”.

#### 3.2.2. Explanatory Variable

The explanatory variable of the study was subjective poverty. Scholars measure subjective poverty mainly through the following measures: the assessment of the minimum income needed to maintain life [53], the assessment of different levels of household income [54], the assessment of the difficulty of maintaining life with actual disposable income [55], and the assessment of individuals’ own economic and social conditions [56,57]. Among these measures, the assessment of an individual’s economic and social conditions is more closely related to the individual’s happiness and subjective welfare, so it is generally considered to be a more comprehensive measure of an individual’s actual feelings on life conditions [58]. It has also been pointed out that this measurement method has the distinct advantage of avoiding many problems in the evaluation of monetary measures [59]. In this study, we used individuals’ self-assessment of their own economic status to evaluate and measure subjective poverty. In the CLHLS 2018 questionnaire, subjective poverty was measured by the following question: How do you rate your own economic status compared with others in local area? This question had five choices: “very rich”, “relatively rich”, “average”, “poor”, and “very poor”. We combined “very rich”, “relatively rich”, and “average” into “no subjective poverty” and combined “relatively poor” and “very poor” into “subjective poverty”. “No subjective poverty” was recorded as 0, and “subjective poverty” was recorded as 1.

#### 3.2.3. Mediating Variables

The mediator in our study was social capital. We divided social capital into two categories: bonding social capital and bridging social capital. In the CLHLS 2018 questionnaire, bonding social capital was measured by the following two questions: (1) To whom do you usually talk most frequently in daily life? (2) To whom do you talk first when you need to tell something of your thoughts? The two questions had eleven identical choices: “spouse”, “son”, “daughter”, “daughter-in-law”, “son-in-law”, “grandchildren and their spouses”, “other relatives”, “friends”, “social workers”, “housekeeper”, and “nobody”. If the respondent chose relatives, friends and housekeeper, the answer was coded as 1, and all other answers were coded as 0. In the CLHLS 2018 questionnaire, bridging social capital was measured by the following question: Are you currently engaged/participating in any of the following activities? The question had three subquestions, which involved participation in outdoor group activities, indoor entertainment activities, and social activities. Each subquestion had five choices: “Almost every day”, “Not every day, but at least once a week”, “Not every week, but at least once a month”, “Not every month, but sometimes”, and “Never”. The responses of participating at least once a month or more were recorded as 1, and all other choices were recorded as 0.

#### 3.2.4. Control Variables

Following the conventional practices in the literature, we chose the following control variables [60,61,62,63]: sex, age, address, education, ethnicity, co-residence, marriage, instrumental activities of daily living (IADL), activities of daily living (ADL), health change, finances, pension, and region.

The descriptive statistics of the variables are presented in Table 1.

#### 3.2.5. Instrumental Variables

Endogeneity is a common problem in econometric models, and using instrumental variables for testing is usually an effective method. There are two requirements for effective instrumental variable selection: (1) the instrumental variable (IV) should be related with the endogenous explanatory (subjective poverty), and (2) the instrumental variable (IV) must be independent from the outcome variable (mental health) [64]. Accordingly, we selected “housing living conditions” as the instrumental variable (IV) to control for endogeneity. In the CLHLS 2018 questionnaire, the “housing living conditions” was measured by the following question: “During the past year, was your home damaged due to leaks, heavy rain, or broken pipes?”. The question had two valid choices: (1): “Yes” (2) “No”. “Yes” was recorded as 1, and “No” was recorded as 0.

### 3.3. Models

#### 3.3.1. Ordered Probit Model

The outcome variable “mental health” was an ordered discrete variable, which is suitable for analysis with an ordered model. In this paper, we chose the ordered probit to estimate the following model:

(1) $$P\left(MH={MH}_{i}|X,\beta \right)=P(MH={MH}_{1}|X_{1},X_{2},X_{3},\ldots ,X_{n})$$

In this and the following models, i refers to the individual in this survey, and MH refers to the mental health of the individual i. In the oprobit model, there is a latent variable ${MH}^{*}$, which can represent the outcome variable MH but cannot be directly observed, and its value was determined using the following model:

(2) $${MH}_{i}^{*}=\beta_{0}{SP}_{i}+X_{0}^{\prime }\gamma_{i}+\mu_{i}$$

In Model (2), ${SP}_{i}$ refers to the subjective poverty self-evaluation of individual i; $\gamma_{i}$ refers to the matrix of the control variables beyond subjective poverty (such as sex, age, ethnicity, education, marriage, etc.); $\beta_{0i}$ and $X_{0i}^{\prime }$ are parameter estimates; and $\mu_{i}$ is the error term.

#### 3.3.2. Mediating Effect

Using the method proposed by Baron and Kenny [65], we constructed Model (3) and Model (4) on the basis of Model (2):

(3) $${SC}_{i}=\beta_{1}{SP}_{i}+X_{1}^{\prime }\gamma_{i}+\omega_{i}$$

(4) $${MH}_{i}^{*}=\beta_{2}{SP}_{i}+{\lambda_{0}SC}_{i}+X_{2}^{\prime }\gamma_{i}+\varphi_{i}$$

In Model (3) and Model (4), ${SP}_{i}$ refers to the subjective poverty self-evaluation of individual i; ${SC}_{i}$ refers to the social capital of individual i; $\gamma_{i}$ refers to the matrix of control variables beyond subjective poverty (such as sex, age, ethnicity, education, marriage, etc.); $\beta_{1}$, $\beta_{2}$, $X_{1}^{\prime }$, $X_{2}^{\prime }$, and $\lambda_{0}$ are parameter estimates; and $\omega_{i}$ and $\varphi_{i}$ are error terms.

#### 3.3.3. Propensity Score Matching (PSM)

To further explore the causal mechanism of mental health, we used propensity score matching and divided the samples into two groups: the treatment group (subjective poverty) and the control group (no subjective poverty). $y_{i}$ is the outcome variable of the mental health of the elderly, in which $y_{i}^{1}$ is the outcome variable for the mental health of the elderly with subjective poverty, and $y_{i}^{0}$ is the outcome variable for the mental health of the elderly with no subjective poverty. First, we calculated the propensity scores. We incorporated relevant variables (sex, age, education, address, marriage, nationality, co-residence, ADL, IADL, health change, pension, finances, region, etc.) into the regression model to guarantee that the ignorability assumption is met. The probit model was used to predict the conditional probability of the elderly who are in subjective poverty entering the treatment group. Second, we performed propensity score matching. We used K-nearest neighbor (KNN) matching, radius matching, kernel matching, and local linear regression (LLR) matching to test the scores’ robustness. Third, to evaluate the matching quality, we used the balance test to ensure that there was no significant difference in the main variables between the matched treatment group (subjective poverty) and the control group (no subjective poverty). Finally, we calculated the average treatment effect on the treated (ATT) by calculating the differences between the two matched groups. The model is as follows:

(5) $$ATT=E\left(y_{i}^{1}\left|X_{i}\right.=1\right)-E(y_{i}^{0}\left|X_{i}\right.=1)$$

#### 3.3.4. Instrumental Variable (IV) Regression

As stated previously, to further check for endogeneity, we used “housing living conditions” as the instrumental variable (IV) and used the methods of two-stage least squares (2SLS) and limited-information maximum likelihood method (LIML) to conduct endogeneity testing. The model is as follows:

(6) $${MH}_{i}^{*}=\beta_{3}{SP}_{i}+{\lambda_{1}IV}_{i}+X_{3}^{\prime }\gamma_{i}+\sigma_{i}$$

In Model (6), ${SP}_{i}$ refers to the subjective poverty self-evaluation of individual i; IV is an instrumental variable that refers to the housing living conditions of individual i; $\gamma_{i}$ refers to the matrix of the control variables beyond subjective poverty (such as sex, age, ethnicity, education, and marriage, etc.); $\beta_{3}$, $\lambda_{1}$, and $X_{3}^{\prime }$ are parameter estimates; and $\sigma_{i}$ is the error term.

## 4. Results

### 4.1. Sample Description

Table 2 summarizes the statistical characteristics of the respondents. The average mental health score for the total sample was 2.166, with a range from 1 to 3. The average mental health score in the subjective poverty group was 1.863, while the average mental health score in the no subjective poverty group was 2.209, which means that the latter had better mental health than the former. The number of elderly people in the subjective poverty group accounted for 12.394% of the total sample, which was roughly the same as the value in the existing studies [4]. The average social capital of the subjective poverty group was 2.211, lower than that of the no subjective poverty group, which was 2.406. The averages of the bonding and bridging social capital scores in the subjective poverty group were 1.842 and 0.368, respectively; these were slightly lower than their corresponding averages. The averages of the bonding and bridging social capital scores in the no subjective poverty group were 1.924 and 0.482, respectively, which were slightly higher than their corresponding averages. The range of years of schooling for the total sample was 0–22, and the average number of years of schooling was 2.521, while its standard deviation was 3.475; this means that there was a certain gap between the individuals in terms of education years. Specifically, the average number of education years for the subjective poverty group was 1.732, lower than the number for the no subjective poverty group, which was 2.633.

### 4.2. Impacts of Subjective Poverty on the Mental Health of the Elderly

Table 3 shows the parameter estimates and the marginal effects of the oprobit regression for the effect of subjective poverty on the mental health of the elderly. The results in column (1) show that subjective poverty had a significant negative impact on the mental health of the elderly without considering the control variables. Column (2) shows that, after adding the control variables, the negative impact of subjective poverty on the mental health of the elderly remained significant. The above results support our first hypothesis, which states that subjective poverty has a significant negative impact on the mental health of the elderly. The results of the marginal effect in column (3) shows that the probabilities of the elderly who experienced subjective poverty and chose “unhealthy” and “not very healthy” increased by 5.3% and 1.6%, respectively, while the probability of those who chose “healthy” decreased by 6.9%.

### 4.3. Heterogeneity Analysis

The above results verified that subjective poverty had a significant negative impact on the mental health of the elderly. However, due to the differences among individuals, the impact of subjective poverty on the mental health of the elderly is inevitably influenced by their idiosyncratic characteristics. The preceding results, at the full sample level, showed the average effect. For further verify Hypothesis 1, we proceeded to investigate the heterogeneity of the impact of subjective poverty on the mental health of the elderly. Using the oprobit regression model, we took the factors of sex, marriage, and regions into consideration, and examined the differences of these factors on the relationship between subjective poverty and the mental health of the elderly. The heterogeneity analysis results are reported in Table 4.

For both male and female seniors, the results for the sex subgroup showed that subjective poverty had a significant negative impact on mental health. Regarding the effect size, the probability of subjective poverty affecting the mental health of elderly females was higher than that for males. In terms of marital status, subjective poverty had a significant negative impact on the mental health of the elderly regardless of whether or not they had a spouse. The results of the heterogeneity analysis regarding marriage were in line with the results for the full sample. In terms of the effect size, subjective poverty had a higher probability of influencing the mental health of the elderly with spouses than that of those without spouses. With regard to the region, apart from the central region, the subgroup results for the eastern and western regions supported Hypothesis 1.

To summarize, the heterogeneity analysis demonstrated that, except for the central region subgroup, all the other subgroups exhibited the same pattern of influence regarding subjective poverty, although the magnitude of such influence did vary from group to group. The results of the heterogeneity analysis provided additional support for the previous results.

### 4.4. Propensity Score Matching (PSM)

To rectify the potential bias in the variable selection process and verify the net effect of subjective poverty on the mental health of the elderly, we used PSM to test for biases. Table 5 shows the PSM results. Using the K-nearest neighbor (KNN) matching, radius matching, kernel matching, and local linear regression (LLR) matching, we calculated the average treatment effect on the treated (ATT). The PSM results were consistent with the previous results. Hypothesis 1, which states that subjective poverty has a significant negative impact on the mental health of the elderly, was again confirmed. To ensure the quality of the PSM, we also conducted a balancing test, and Table 6 shows the results. The standardization error of all the variables, after matching, was less than 10%. In addition, except for only a few samples, the vast majority of samples supported Hypothesis 1, and the t-value results obtained using all of the matching methods in this study supported the null hypothesis that all the covariates had no systematic bias between the treatment group and the control group. Therefore, the PSM test further confirmed the robustness and reliability of the results.

### 4.5. Instrumental Variable Test

To summarize, our foregoing results demonstrated that subjective poverty had a significant negative impact on the mental health of the elderly; however, there could be endogeneity between the mental health and subjective poverty. In order to eliminate potential endogenous bias in the model, we chose “housing living conditions” as an instrumental variable and used the two-stage least-squares (2SLS) and limited information maximum likelihood (LIML) to conduct endogeneity testing. The instrumental variable regression results are reported in Table 7. The results showed that there was no difference in the estimated coefficient values between the 2SLS and the LIML. In addition, the results of the first-stage regression showed that the F-value was significant. At 13.556, it exceeded the empirical cutoff point of 10. This means that the impact of the instrumental variable on subjective poverty was statistically significant, and the selected instrumental variable was not a weak instrumental variable of subjective poverty. The results of the second-stage regression also showed that subjective poverty had a significant impact on the mental health of the elderly. We are therefore confident that the oprobit results are robust and reliable.

### 4.6. The Mediating Effect of Social Capital

Finally, we considered the mediating effect of social capital. The estimated results of such effect are shown in Table 8. Column (1) shows that subjective poverty had a significant negative effect on mental health. Column (2) shows that there was a significant negative correlation between subjective poverty and social capital with the control variables. When both subjective poverty and social capital were included in the model, column (3) shows that the negative impact of subjective poverty on mental health remained significant. The effect size of social capital on mental health was 0.085, and the *p*-value was significant at the level of 1%, which means that social capital had a positive impact on the mental health of the elderly. The results in columns (1), (2), and (3) all met the conditions for the mediating effect, and strongly supported Hypothesis 2; namely, social capital partially mediates the relationship between subjective poverty and mental health among the elderly.

Column (4) shows that subjective poverty had a statistically significant impact on bonding social capital. Column (5) shows that, when subjective poverty and bonding social capital were included in the model, there was a significant negative correlation between subjective poverty and mental health. It also shows that the bonding social capital had a significant positive impact on the mental health of the elderly. In summary, columns (1), (4), and (5) strongly supported Hypothesis H2a, i.e., bonding social capital partially mediates the relationship between subjective poverty and mental health among the elderly. Column (6) shows that the impact of subjective poverty on bridging social capital was significantly negative at the 10% level. Column (7) shows that the effect sizes of subjective poverty and bridging social capital on mental health were −0.199 and 0.079, respectively, and their *p*-values were significant at the 1% level. Considering the results in columns (1), (6), and (7), we can conclude that bridging social capital also had a partial mediating effect in the relationship between subjective poverty and mental health, and Hypothesis H2b was supported by the empirical results.

## 5. Discussion

In this study, using data from CLHLS 2018, we explored the impact of subjective poverty on the mental health among the elderly in China and considered the mediating effect of social capital. Our results confirm that subjective poverty has a significant negative impact on mental health, and that social capital partially mediates the relationship between subjective poverty and mental health. We categorized social capital into two types: bonding and bridging social capital. The results show that, as mediators, bonding and bridging social capital partially mediate the relationship between subjective poverty and mental health. This study provides new evidence for the relationship between subjective poverty and mental health among the elderly in low- and middle-income countries and also confirms the underlying mechanism of social capital in the relationship between subjective poverty and mental health.

Our findings provide additional support to conclusions reached by many existing studies. For example, Xu et al. found that the relationship between subjective poverty and higher mortality among the elderly was partially mediated by mental health [66]. Ervin et al. found that, in Australia, compared with those in relative non-poverty, older women in relative poverty often had poorer mental health [67]. Ayalon et al. found that elderly people in subjective poverty in Israel were more likely to suffer from loneliness [22]. As is known, the mental health of individuals is affected by their surrounding environment [68], and the problems caused by subjective poverty can not only worsen the surrounding environment of individuals but also have negative effects, such as relative deprivation, shame, and pressure [69], which can lead to the loss of psychological resources.

We examined the impacts of subjective poverty on mental health at different gradient levels. To our knowledge, this was the first attempt to study the relationship between subjective poverty and the mental health of the elderly. We found that the elderly who believed they were poor often scored worse at all gradient levels of mental health than those who believed they were not poor. We also found that there were differences in the probabilities of subjective poverty affecting mental health at different gradient levels. Our results report that, for the elderly in subjective poverty, the probability of choosing “unhealthy” and “not very healthy” increased by 5.3% and 1.6%, respectively, while the probability of choosing “healthy” decreased by 6.9%. This conclusion is an interesting discovery that deserves further exploration in the future.

Our study also revealed that social capital, whether bonding social capital or bridging social capital, had a positive effect on the mental health. The positive effects of bonding social capital on the mental health of the elderly have been confirmed in many studies [70]. Nyqvist et al. observed that, for the elderly, close relationships can provide trust, support, and a sense of security, which are greatly beneficial to the mental health [33]. Compared to bonding social capital, the relationship between bridging social capital and mental health often had contrasting conclusions [41]. We found that bridging social capital, comprising outdoor group activities, indoor entertainment activities, participation in social activities, etc., could promote the mental health of the elderly. The positive impact of social capital, particularly bonding and bridging social capital, on the mental health of the elderly encourages us to explore further how to improve China’s policy interventions to tackle the aging issue. This can also extend our discussion as to how to better formulate health policies for the elderly in low- and middle-income countries.

In the mediating effect analysis, we confirmed that social capital, bonding social capital, and bridging social capital all had partial mediating effects in the relationship between subjective poverty and mental health. It is useful to explore the mechanism of social capital in the study of the relationship between subjective poverty and mental health. Our mediating effect results indicate that the impact of subjective poverty on mental health is partially achieved through the path of social capital (including bonding and bridging social capital), and that the weakening of the social capital (including bonding and bridging social capital) of the elderly is an important reason why subjective poverty affects mental health negatively and leads to poor mental health outcomes. On the one hand, subjective poverty can cause individuals to lose the feeling of relevance and importance in their intimate relationships [71], damage their decision-making capabilities and self-esteem, and even lead to them being blamed and criticized for their relatively weak position with respect to economic and social aspects [72]. On the other hand, subjective poverty can make individuals feel more indifferent to and alienated from the process of interacting with outsiders and can generate a stronger sense of discrimination and social exclusion, which greatly increases the possibility of psychological distress [73]. Based on our mediating effect results, we can conclude that social capital acts as a “pressure-reducing valve” between subjective poverty and mental health and that it can, to a certain extent, cushion the negative effects of subjective poverty on the mental health of the elderly.

In our heterogeneity analysis, we first considered sex. We found that subjective poverty had a higher probability of affecting the mental health of elderly females than of elderly males. One reason for this could be that females may be more sensitive to their surroundings than males [74,75]. In addition, long-standing gender inequality in society and culture has led women to be under more pressure than men in families, daily life, and work [67]. As for marital status, we found that subjective poverty had a greater impact on the mental health of the elderly who have a spouse than those without a spouse. One study has shown that individuals’ perceptions of their economic and social status influence their expectations and reactions to their spouses, which further affects their quality of life of their family [76]. Elderly people in subjective poverty may encounter more resource tension and conflicts with their spouses in life, which could have a greater negative effect on mental health. Another consideration in the heterogeneity analysis was region. In this study, the results for the east and west regions showed that subjective poverty had a significant negative impact on the mental health of the elderly, but the results for the central region did not support this conclusion. In terms of income, personal wealth, and the wealth gap, there is a significant gap between the eastern and western regions, while the central region is relatively balanced; this may be the main reason why the results in the central region were not significant.

There were some limitations in our study. First, we used cross-sectional data, and although this is common in studies, the causal relationship between subjective poverty and mental health still needs to be treated with caution. Second, there may be reverse causality between subjective poverty and mental health, which may lead to bias in the estimates. In our study, we used instrumental variable regression and tested the robustness of our conclusions. However, the effect of reverse causation can not be entirely eliminated because subjective poverty and mental health do affect each other in both ways. We will further tackle this issue in our future research. Third, the core variables of subjective poverty, mental health, and social capital used in our study were obtained through self-reporting, which inevitably leads to social expectation bias; future studies can adopt multiple measurement methods to eliminate this bias. Fourth, the influencing factors of mental health are extremely complex. Following previous studies, we included all the conventional control variables in the model as they are available. However, there still can exist some confounding factors that were not controlled. Finally, there may be some errors in variable measurement, and more comprehensive measurement scales should be developed and used in the future to improve the scientific and accurate measurement of the variables.

## 6. Conclusions

The study evaluated the impact of subjective poverty on the mental health among the elderly in China and the mediating role of social capital. We found that subjective poverty has a significant negative impact on mental health, and social capital (including bonding and bridging social capital) plays a partial mediating role in the relationship between subjective poverty and mental health. Our findings also suggested that social capital, to a certain extent, could cushion the negative effects of subjective poverty on mental health. We believe that, in the process of implementing the government’s healthy aging strategy in China, more importance should be attached to the subjective poverty of the elderly. Strengthening the construction of social networks that have bonding and bridging social capital as the core, in the new era, could be an important method of coping with subjective poverty and safeguarding the mental health and wellbeing of the elderly.

## Figures and Tables

**Table 1:** Variable descriptions and codes.

Variable	Variable Description	Codes
Mental health	Respondent’s mental health status	1 = Unhealthy; 2 = Not very healthy; 3 = Healthy
Subjective poverty	Respondent’s self-evaluation of their own economic situation compared to others in the local environment	0 = No; 1 = Yes
Social capital	Sum of the scores of the bonding and bridging social capital	The scores range from 0 to 5; the higher the score, the more social capital
Bonding social capital	Respondent’s score regarding who they talked to the most and who they talked to first	The scores range from 0 to 2; the higher the score, the more bonding social capital
Bridging social capital	Respondent’s score with respect to participation in outdoor group activities, indoor recreational activities, and social activities	The scores range from 0 to 3; the higher the score, the more bridging social capital
Sex	Respondent’s sex	0 = Male; 1 = Female
Age	Respondent’s age	Calculated by 2018 minus the respondent’s birth year
Ethnicity	Whether the respondent is Han or an ethnic minority	0 = Han; 1 = Minority
Education	Respondent’s years of schooling	The years range from 0 to 22; the higher the number of years, the more education they received
Marriage	Whether the respondent has a spouse	0 = Have a spouse; 1 = No spouse
Address	Respondents’ residence	0 = City/Town; 1 = Rural
Co-residence	Who the respondent lives with	1 = With household member(s); 2 = Alone; 3 = In a nursing home
ADL	Respondent’s score for six activities of daily living (including bathing, dressing, toileting, indoor transfer, continence, and eating in the past six months)	1 = Unable to care for self; 2 = Partial self-care; 3 = Able to care for self
IADL	Respondent’s score for six instrumental activities of daily living (including visiting, shopping, cooking, laundry, transportation, etc.)	1 = Unable to care for self; 2 = Partial self-care; 3 = Able to care for self
Health change	Respondent’s rating of their present health compared to one year ago	0 = Unchanged or better; 1 = Worse
Finances	Respondent’s financial ability to pay daily costs	0 = Yes; 1 = No
Pension	Whether the respondent has commercial pension	0 = Yes; 1 = No
Region	Where the respondent lives in China	0 = East; 1 = Central; 2 = West

**Table 2:** Sample characteristics (*N* = 8811).

Variable	Total	Subjective Poverty	No Subjective Poverty	
	Mean (SD)	Mean (SD)	Mean (SD)	Range
Mental Health	2.166 (0.762)	1.863 (0.756)	2.209 (0.753)	1–3
Subjective Poverty	0.124 (0.330)	1.000 (0.000)	0.000 (0.000)	0–1
Social Capital	2.382 (0.794)	2.211 (0.808)	2.406 (0.789)	0–5
Bonding Capital	1.914 (0.360)	1.842 (0.489)	1.924 (0.336)	0–2
Bridging Capital	0.468 (0.692)	0.368 (0.618)	0.482 (0.700)	0–3
Sex	0.588 (0.492)	0.598 (0.490)	0.587 (0.492)	0–1
Age	84.802 (11.768)	84.725 (11.753)	84.813 (11.771)	65–117
Age^2^	7329.848 (2010.287)	7316.372 (2015.799)	7331.755 (2009.629)	4225–13,689
Education	2.521 (3.475)	1.732 (2.788)	2.633 (3.548)	0–22
Address	0.497 (0.500)	0.549 (0.498)	0.489 (0.500)	0–1
Marriage	0.585 (0.493)	0.614 (0.487)	0.581 (0.493)	0–1
Ethnicity	0.934 (0.248)	0.923 (0.267)	0.936 (0.245)	0–1
Co-residence	1.218 (0.469)	1.284 (0.497)	1.209 (0.464)	1–3
ADL	17.069 (2.231)	16.750 (2.715)	17.114 (2.150)	6–18
IADL	11.978 (3.758)	11.616 (3.900)	12.029 (3.734)	5–15
Health change	0.364 (0.481)	0.531 (0.499)	0.340 (0.474)	0–1
Pension	0.373 (0.484)	0.342 (0.474)	0.377 (0.485)	0–1
Finances	0.160 (0.367)	0.637 (0.482)	0.093 (0.290)	0–1
Region	0.838 (0.842)	0.933 (0.836)	0.824 (0.843)	0–2

**Table 3:** Impacts of subjective poverty on the mental health of the elderly (*N* = 8811).

Variable	(1)	(2)	(3) Marginal Effects (dy/dx)		
	Oprobit	Oprobit	Unhealthy	Not Very Healthy	Healthy
Subjective poverty	−0.637 ***	−0.201 ***	0.053 ***	0.016 ***	−0.069 ***
	(0.028)	(0.042)	(0.011)	(0.003)	(0.014)
Sex		−0.062 **	0.016 **	0.005 **	−0.021 **
		(0.028)	(0.007)	(0.002)	(0.009)
Age		0.010	−0.003	−0.001	0.004
		(0.017)	(0.004)	(0.001)	(0.006)
Age^2^		−0.000	0.000	0.000	−0.000
		(0.000)	(0.000)	(0.000)	(0.000)
Education		0.036 ***	−0.009 ***	−0.003 ***	0.012 ***
		(0.004)	(0.001)	(0.000)	(0.001)
Residence		−0.015	0.004	0.001	−0.005
		(0.025)	(0.007)	(0.002)	(0.009)
Marriage		−0.083 **	0.022 **	0.007 **	−0.028 **
		(0.032)	(0.008)	(0.003)	(0.011)
Ethnicity		0.138 ***	−0.036 ***	−0.011 ***	0.047 ***
		(0.049)	(0.013)	(0.004)	(0.017)
Co-residence		−0.126 ***	0.033 ***	0.010 ***	−0.043 ***
		(0.027)	(0.007)	(0.002)	(0.009)
ADL		0.046 ***	−0.012 ***	−0.004 ***	0.016 ***
		(0.008)	(0.002)	(0.001)	(0.003)
IADL		0.047 ***	−0.012 ***	−0.004 ***	0.016 ***
		(0.005)	(0.001)	(0.000)	(0.002)
Health change		−0.367 ***	0.096 ***	0.030 ***	−0.126 ***
		(0.026)	(0.007)	(0.002)	(0.009)
Pension		0.131 ***	−0.034 ***	−0.011 ***	0.045 ***
		(0.026)	(0.007)	(0.002)	(0.009)
Finances		0.351 ***	0.092 ***	0.029 ***	−0.120 ***
		(0.038)	(0.010)	(0.003)	(0.013)
Central		0.090 ***	0.024 ***	0.007 ***	−0.031 ***
		(0.030)	(0.008)	(0.002)	(0.010)
West		0.105 ***	0.028 ***	0.009 ***	−0.036 ***
		(0.031)	(0.008)	(0.003)	(0.010)
*N*	15,750	8811	8811	8811	8811
Pseudo R^2^	0.016	0.083			

Note: ** *p* < 0.05, *** *p* < 0.01; robust standard errors in parentheses; dy/dx for factor levels is the discrete change from the base level; columns (1) and (2) report the parameter regression coefficients, while column (3) reports the marginal effects.

**Table 4:** Heterogeneity analysis (dy/dx) (*N* = 8811).

Variable	Subgroup: Sex		Subgroup: Spouse		Subgroup: Region		
	(1) Male	(2) Female	(3) Yes	(4) No	(5) East	(6) Central	(7) West
Subjective poverty	−0.138 **	−0.248 ***	−0.272 ***	−0.154 ***	−0.213 ***	−0.111	−0.270 ***
	(0.067)	(0.054)	(0.068)	(0.053)	(0.070)	(0.080)	(0.070)
Control	Yes	Yes	Yes	Yes	Yes	Yes	Yes
*N*	3627	5184	3655	5156	3957	2326	2528
Pseudo R^2^	0.080	0.077	Yes	Yes	0.086	0.087	0.082

Note: ** *p* < 0.05, *** *p* < 0.01; robust standard errors in parentheses; dy/dx for factor levels is the discrete change from the base level; column (1) to column (7) report the oprobit regression coefficients for subjective poverty.

**Table 5:** Propensity score matching results.

Methods	Sample	Subjective Poverty = (1)	No Subjective Poverty = (2)	ATT = (1) − (2)	S.E.	*t*-Value
KNN (K = 4)	Unmatched	1.863	2.209	−0.347	0.024	−14.24 ***
	Matched	1.864	1.979	−0.115	0.033	−3.50 ***
Radius Matching (c = 0.01)	Unmatched	1.863	2.209	−0.347	0.024	−14.24 ***
	Matched	1.864	1.978	−0.114	0.033	−3.48 ***
Kernel Matching	Unmatched	1.863	2.209	−0.347	0.024	−14.24 ***
	Matched	1.864	2.017	−0.153	0.030	−5.12 ***
Local Linear Regression Matching	Unmatched	1.863	2.209	−0.347	0.024	−14.24 ***
	Matched	1.864	1.991	−0.127	0.041	−3.11 ***

Note: *** *p* < 0.01.

**Table 6:** Balancing test results based on the propensity score matching.

Methods	Sample	Ps R^2^	LR chi^2^	*p* > chi^2^	Mean Bias	Med Bias
KNN (K = 4)	Unmatched	0.259	1707.56	0.000	19.7	8.6
	Matched	0.003	8.89	0.883	2.6	1.6
Radius Matching (c = 0.01)	Unmatched	0.259	1707.56	0.000	19.7	8.6
	Matched	0.003	8.80	0.888	2.5	1.4
Kernel Matching	Unmatched	0.259	1707.56	0.000	19.7	8.6
	Matched	0.005	13.68	0.550	3.6	2.0
Local Linear Regression Matching	Unmatched	0.259	1707.56	0.000	19.7	8.6
	Matched	0.004	13.21	0.586	2.7	2.2

**Table 7:** Instrumental variable regression results.

Variable	(1) 2SLS		(2) LIML	
	First Stage	Second Stage	First Stage	Second Stage
Subjective Poverty		−1.369 **		−1.369 **
		(0.665)		(0.665)
IV	0.034 ***		0.034 ***	
	(0.009)		(0.009)	
Control Variable	Yes	Yes		Yes
*N*	8562	8562	8562	8562
F	13.556		13.556	
Pseudo R^2^/R^2^	0.255		0.255	

Note: ** *p* < 0.05, *** *p* < 0.01; robust standard errors in parentheses.

**Table 8:** Mediating effect of social capital (dy/dx).

Variable	Oprobit						
	(1)	(2)	(3)	(4)	(5)	(6)	(7)
	Mental Health	Social Capital	Mental Health	Bonding Social Capital	Mental Health	Bridging Social Capital	Mental Health
Subjective Poverty	−0.201 ***	−0.166 ***	−0.193 ***	−0.296 ***	−0.195 ***	−0.090 *	−0.199 ***
	(0.042)	(0.046)	(0.042)	(0.070)	(0.042)	(0.050)	(0.042)
Social Capital			0.085 ***				
			(0.017)				
Bonding Social Capital					0.109 ***		
					(0.037)		
Bridging Social Capital							0.079 ***
							(0.019)
Control Variables	Yes	Yes	Yes	Yes	Yes	Yes	Yes
*N*	8811	8811	8811	8811	8811	8811	8811
Pseudo R^2^	0.083	0.081	0.085	0.080	0.084	0.102	0.084

Note: * *p* < 0.1, *** *p* < 0.01; robust standard errors in parentheses; dy/dx for factor levels is the discrete change from the base level; column (1) to column (7) report the oprobit regression coefficients.

## Data Availability

Datasets are distributable only by the CLHLS team. They are available in the public domain on the CLHLS website, https://opendata.pku.edu.cn/dataverse/CHADS (accessed on 9 September 2022), and are also available on request from the corresponding author.
